# Do physical activity interventions in Indigenous people in Australia and New Zealand improve activity levels and health outcomes? A systematic review

**DOI:** 10.1186/s12966-016-0455-x

**Published:** 2016-12-21

**Authors:** Ashleigh Sushames, Jannique G.Z. van Uffelen, Klaus Gebel

**Affiliations:** 1 College of Healthcare Sciences, James Cook University, Cairns, QLD Australia; 2 Centre for Chronic Disease Prevention, College of Public Health, Medical and Veterinary Sciences, James Cook University, 14-88 McGregor Road, Smithfield, Cairns, QLD 4878 Australia; 3 Institute of Sport, Exercise and Active Living, Victoria University, Footscray Park Campus, Melbourne, VIC 3000 Australia; 4 Department of Kinesiology, Physical Activity, Sports and Health Research Group, KU Leuven - University of Leuven, Leuven, B-3000 Belgium; 5 School of Allied Health, Faculty of Health Sciences, Australian Catholic University, North Sydney, NSW Australia; 6 Prevention Research Collaboration, Sydney School of Public Health, University of Sydney, Sydney, NSW Australia

**Keywords:** Indigenous health, Australia, New Zealand, Physical activity, Intervention

## Abstract

**Background:**

Indigenous Australians and New Zealanders have a significantly shorter life expectancy than non-Indigenous people, mainly due to differences in prevalence of chronic diseases. Physical activity helps in the prevention and management of chronic diseases, however, activity levels are lower in Indigenous than in non-Indigenous people.

**Objective:**

To synthesise the literature on the effects of physical activity interventions for Indigenous people in Australia and New Zealand on activity levels and health outcomes.

**Methods:**

The Cochrane Library, MEDLINE, SPORTSDiscus and PsycINFO were searched for peer-reviewed articles and grey literature was searched. Interventions targeted Indigenous people in Australia or New Zealand aged 18+ years and their primary or secondary aim was to increase activity levels. Data were extracted by one author and verified by another. Risk of bias was assessed independently by two authors. Data were synthesised narratively.

**Results:**

407 records were screened and 13 studies included. Interventions included individual and group based exercise programs and community lifestyle interventions of four weeks to two years. Six studies assessed physical activity via subjective (*n* = 4) or objective (*n* = 2) measures, with significant improvements in one study. Weight and BMI were assessed in all but one study, with significant reductions reported in seven of 12 studies. All five studies that used fitness tests reported improvements, as did four out of eight measuring blood pressure and seven out of nine in clinical markers.

**Conclusions:**

There was no clear evidence for an effect of physical activity interventions on activity levels, however, there were positive effects on activity related fitness and health outcomes.

**Trial Registration:**

The review protocol was registered with PROSPERO (registration number: CRD42015016915).

## Background

Internationally, Indigenous people have disproportionately higher rates of preventable chronic disease that contribute to a lower life expectancy than non-Indigenous people [1–3]. Australia and New Zealand are both First World countries with substantial inequalities in health status between Indigenous and non-Indigenous people. In Australia, Aboriginal and Torres Strait Islanders are the Indigenous people, accounting for 3% of the population [4], and their life expectancy is 10 years lower than that of non-Indigenous Australians [5]. In New Zealand, the Māori people are the Indigenous custodians, accounting for 15.6% of the population [6], and their life expectancy is 7.1 years lower than among the non-Indigenous population [7].

There is a variety of complex and interrelated social, economic and historical factors that contribute to premature death in Indigenous populations [8]. In Australia, it is estimated that 70% of the inequalities in health are caused by chronic disease [9]. Obesity related lifestyle diseases, such as type 2 diabetes and cardiovascular disease, are some of the leading causes of morbidity and mortality in Indigenous populations in Australia and New Zealand [5, 10]. For instance, the age-standardised mortality rate for type 2 diabetes in Indigenous Australians is nearly five times higher than in other Australians [11], and four times higher in Māori people compared to other New Zealanders [12]. The age-adjusted death rate for Indigenous adults from cardiovascular disease is almost twice that of other people in Australia [13] and New Zealand [6].

Excess body fat is a modifiable precursor of type 2 diabetes and cardiovascular disease [14]. In Australia, 66% of Aboriginal and Torres Strait Islander people over 15 years of age are overweight or obese, more than among non-Indigenous Australians (rate ratio of 1.1) [15]. In New Zealand obesity rates for adults are estimated to be 46% for Māoris (rate ratio of 1.8 compared to non-Māori) [16]. There is substantial evidence for the benefits of physical activity in preventing, managing and improving conditions such as obesity, type 2 diabetes and cardiovascular disease [17, 18]. For example, regular physical activity can increase insulin sensitivity both acutely and long term, lower blood sugar levels, reduce body fat and improve cardiovascular function [19]. Current physical activity guidelines recommend that adults aged 18 to 64 should accumulate 150–300 minutes per week of moderate physical activity or 75–150 minutes of vigorous activity [20]. However, in Australia, only 38% of Indigenous people in non-remote locations meet the physical activity recommendations. Furthermore, compared to non-Indigenous Australians, Indigenous people in Australia are less likely to be sufficiently active for health (rate ratio 0.8) and to participate in any physical activity (rate ratio 0.9) [21]. In New Zealand 50.1% of Māoris are sufficiently active compared to 51% in the overall population [16].

Physical activity interventions have proved effective to increase activity levels in the general population [22, 23], however, these studies usually only include a very small percentage of Indigenous people and results are not reported separately [24, 25]. Moreover, general population interventions may not always be appropriate for Indigenous population groups. Cultural adaptations should be incorporated to ensure that the interventions adhere to the community values and beliefs. This is due to Indigenous populations being a minority and diverse group that, due to cultural differences, need specific consideration when designing programs. Recommendations have previously been made by organisations, such as the National Health and Medical Research Council in Australia, providing a “Road Map” in regards to working with Indigenous populations and the cultural factors that researchers need to consider before designing programs [26].

A recent literature review aimed to synthesise the evidence on the effects of group-based sport and exercise programs for Indigenous adults on anthropometric and physiological outcomes and quality of life [27]. However, although this review was described as ‘systematic’, the authors of this review, which synthesised six studies, omitted several studies that would have been eligible for inclusion in our view. Moreover, this review did not meet all the criteria of a systematic review, such as searching for grey literature, and the authors did not follow the PRISMA statement [28], nor did they report sufficient information on the methodology, such as search terms, to make the review replicable. A systematic review, including 64 studies, by Teufel-Shone and colleagues on Indigenous people in the United States and Canada found physical activity interventions to be effective on health outcomes related to physical activity such as fitness and blood pressure [29]. Some reviews have synthesised the findings of different kinds of interventions targeting chronic disease or risk factors for disease in Indigenous Australians and New Zealanders, however, these reviews were not specifically focused on physical activity interventions [30]. A literature review by Clifford and colleagues identified 20 interventions which addressed smoking, nutrition, alcohol misuse, or physical inactivity in Australian Indigenous populations [31]. However, only five of the interventions had a specific focus on physical activity, and this review did not summarise results of the interventions as its main objective was to describe and critique the methodology of the interventions. Overall, there is a lack of knowledge on the effect of physical activity interventions on activity levels and health outcomes in Indigenous people residing in Australia and New Zealand.

Given the gaps in the literature and the high prevalence of chronic disease and physical inactivity in Indigenous people in Australia and New Zealand and the important role that physical activity has in the prevention and management of these chronic diseases, a systematic review of effectiveness of interventions to promote activity in Indigenous populations is warranted. This systematic review fills significant gaps in the literature by identifying, critically appraising and synthesising the evidence on the effects of physical activity interventions designed for Indigenous people in Australia and New Zealand on activity levels and health outcomes. As well, we provide recommendations for future research in physical activity interventions for Indigenous people.

## Methods

### Literature Search

The PROSPERO database and the Cochrane Library were searched for systematic reviews on physical activity interventions designed for Indigenous people in Australia or New Zealand. Once it was determined that there were no such reviews published or in progress, the review protocol was registered with PROSPERO, the international prospective register for systematic reviews (registration number: CRD42015016915) [32]. This review follows the PRISMA statement for systematic reviews [28].

**Fig. 1:**
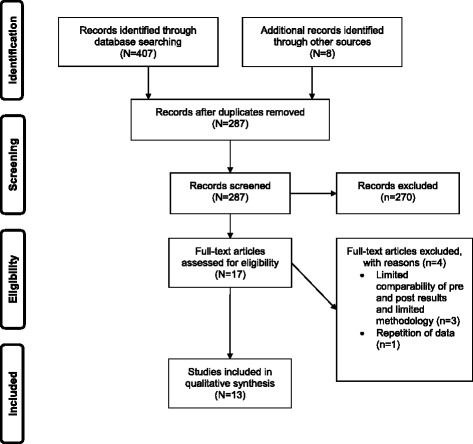
Flowchart of the selection process for inclusion of articles in the systematic review

The Cochrane Library, MEDLINE, SPORTSDiscus and PsycINFO were searched from their start dates until the 14th of March 2016 for physical activity interventions for Indigenous people in Australia and New Zealand. Search terms for population were ‘Oceanic ancestry group’ OR ‘population groups’ OR ‘Aborig*’ OR ‘Indigenous*’ and were combined with ‘intervention*’ and physical activity related terms (‘exercise’ OR ‘sports’ OR ‘motor activity’ OR ‘physical*’) and the free terms ‘physical*’ OR ‘fitness*’. No restrictions on language or publication years were applied. We undertook forward and backward citation tracking from the identified papers. Government websites and databases were searched for grey literature. Health departments were e-mailed in search of interventions that may not have been identified. Experts in the field were contacted to search for additional references. Search results and screening outcomes are presented in a flow-diagram (Fig. 1).

### Study Inclusion and Exclusion Criteria

To be included in the review studies had to evaluate an intervention with a physical activity component for Indigenous people residing in Australia or New Zealand aged 18 years or over. Indigenous groups included Aboriginal people and Torres Strait Islanders in Australia and the Māori people in New Zealand. While it is acknowledged that Pacific Islander people are not Indigenous to New Zealand, there are historical and cultural connections with Māori people [33]. Therefore, studies conducted in New Zealand with Pacific Islander communities were also considered eligible for inclusion into the review. Physical activity is a broad term which encompasses different forms of body movement produced by skeletal muscle, resulting in energy expenditure above resting level [34]. This includes exercise, sport and incidental physical activity. Studies with multi-component interventions were included if increasing physical activity levels was a core component of the intervention either through exercise programs or health education aiming to promote activity. Ideally the interventions would include a non-exposed control group. However, it has been found to be difficult to recruit participants for control groups in Indigenous communities [35, 36]. Therefore, uncontrolled studies with rigorous protocols were included. Protocols were deemed as rigorous if sufficient methodological details of the program were provided to allow for replication, and if the pre and post outcome measures were directly comparable. The studies had to measure physical activity and/or activity related health outcomes before and after the intervention. Studies were excluded if they did not specifically target Indigenous people, but only included them as sub groups.

### Data Extraction and Analysis

Two authors (AS and KG) independently screened the articles found in the literature search and excluded those that did not meet the inclusion criteria based on title, abstract and full text. Differences in reviewers’ decisions were resolved through discussion and consensus. Data were extracted by AS and cross-checked by KG. A standardised abstraction procedure based on Zaza et al. [37] was used to extract data from the included studies. Information extracted included study population, study setting (such as remote/rural), as well as demographic and baseline characteristics of the participants. Details of the intervention and control conditions along with the study methodology, recruitment strategy, retention rates, physical activity and activity related health outcomes, the timing of measurements, and the acceptability of the program to the Indigenous community were also extracted.

### Appraisal of Study Quality

Two authors (AS and KG) independently appraised the quality of the included studies with the Quality Assessment Tool for Quantitative Studies [38] (Table 1). The tool is used to rate several aspects of studies, such as study design, selection bias, retention rates and data collection methods, as well as the study overall as strong, moderate or weak. Differences in ratings of study quality were resolved through discussion and consensus.

### Data Synthesis

Because of the heterogeneity of the study designs, predictor and outcome variables and the contexts in which the data were collected, a meta-analysis was not possible. Therefore, a narrative synthesis of the evidence was conducted. The data synthesis contains findings from the included studies such as type of intervention, target population characteristics (Table 2), types of outcomes that were assessed and the duration of the intervention (Table 3). The effect of the intervention in each study is summarised and it is noted whether changes were significant, and what direction the changes were in (positive or negative).

**Table 1:** Quality rating scores for all papers included in the systematic review._NA=Not applicable_

Author	Selection bias	Study design	Confounders	Blinding	Data collection method	Withdrawals and dropouts	Overall rating
Biddle et al. [48]	Weak	Strong	Weak	NA	Strong	Strong	Weak
Canuto et al. [49]	Moderate	Strong	Strong	NA	Strong	Weak	Moderate
Chan et al. [43]	Weak	Moderate	NA	NA	Strong	Moderate	Moderate
Coppell et al. [50]	Weak	Moderate	NA	Weak	Strong	Weak	Weak
Davey et al. [40]	Weak	Moderate	NA	NA	Strong	Moderate	Moderate
Dimer et al. [51]	Moderate	Moderate	NA	NA	Strong	Weak	Moderate
Egger et al. [42]	Weak	Strong	NA	NA	Strong	Strong	Moderate
Gracey et al. [52]	Strong	Moderate	NA	NA	Strong	Weak	Moderate
McAuley et al. [36]	Weak	Moderate	NA	NA	Strong	Strong	Moderate
Mendham et al. [41]	Moderate	Strong	Strong	Moderate	Strong	Weak	Moderate
O’Dea [39]	Weak	Moderate	NA	NA	Strong	Strong	Moderate
Rowley et al. [35]	Weak	Moderate	Strong	Weak	Strong	Weak	Weak
Sukala et al. [53]	Moderate	Moderate	Strong	Moderate	Strong	Moderate	Strong
Overall score	1 strong, 3 moderate, 9 weak	4 strong, 9 moderate, 0 weak	4 strong, 0 moderate, 1 weak, 8 NA	0 strong, 2 moderate, 2 weak, 9 NA	13 strong, 0 moderate, 0 weak	4 strong, 3 moderate, 6 weak	1 strong, 9 moderate, 3 weak

**Table 2:** Description of study, participants, intervention and retention rates

Study	Ethnicity, location; Study design and size; Participant characteristics and age.	Study title and aim	Intervention	Study retention rates
Biddle et al. [48]	Pacific Islanders, Auckland, New Zealand; RCT, *N* = 20 (control *n* = 9, intervention *n* = 11); Mixed genders, age: 34.8 ± 12.6 years.	Randomised controlled trial of informal team sports for cardiorespiratory fitness and health benefits in Pacific adults. Assess the effectiveness of small-sided games-based exercise on fitness and health parameters among Pacific adults over four weeks.	3 × 45 minute training sessions per week, consisting of small-sided games such as volleyball, touch rugby and cricket.	Intervention: 82% Control: 78%
Canuto et al. [49]	Indigenous Australians, Adelaide, Australia; RCT, *N* = 101 (control *n* = 49, intervention *n* = 51); All female, age: Control: 40.7 years (CI: 37.7–43.6), Intervention: 39.8 (CI: 36.7–43.1).	Pragmatic randomised trial of a 12-week exercise and nutrition program for Aboriginal and Torres Strait Islander women: clinical results immediate post and 3 months follow-up. Evaluate the effectiveness of a 12 week structured exercise and nutrition program.	Structured 12 week group fitness program including exercise classes, incidental activity and walking and nutrition workshops.	Intervention: 57% Control: 61%
Chan et al. [43]	Indigenous Australians, urban Queensland, Australia; Cohort, *N* = 101 (females *n* = 69, males *n* = 63); Mixed gender, age: Diabetics: 56.5 years (52.8–60.1), Non-diabetics: 43 years (39–47).	Short-term efficacy of a lifestyle intervention programme on cardiovascular health outcome in overweight Indigenous Australians with and without type 2 diabetes mellitus: The healthy lifestyle programme (HELP). Determine the effectiveness of lifestyle intervention on improving diabetes and cardiovascular risk factors.	The lifestyle intervention was a community based education program including self-monitoring of fasting glucose and monitoring of physical activity with a pedometer.	80%
Coppell et al. [50]	Māori, rural East Coast of New Zealand; Interrupted time-series, *n* = 286 (Survey 1), *n* = 236 (Survey 2); Mixed genders, age survey 1: 50.25 years (female), 51.4 years (male), survey 2: 49.35 years (female), 50.95 years (male)	Two-year results from a community-wide diabetes prevention intervention in a high risk indigenous community: the Ngati and Healthy project. To reduce the prevalence of insulin resistance in a high risk community.	Local community health promotion programs, a community education program for high risk individuals including cooking classes, exercise class and opportunistic weigh ins.	Survey 1: 48.5% Survey 2: 47.7%
Davey et al. [40]	Aboriginal Australians, Tasmania, Australia; Cohort, *n* = 92 (females *n* = 56, males *n* = 36); Mixed genders, age: ≤ 49 (*n* = 35), 50–59 years (*n* = 35), >60 years (*n* = 22).	Tasmanian Aborigines step up to health: evaluation of a cardiopulmonary rehabilitation and secondary prevention program. To create an ongoing sustainable program of direct benefit to participants and promote the benefits of physical activity to other Aboriginal health service programs and the wider Aboriginal community.	8-week program consisting of two supervised exercise sessions (1hour) and one educational session (1 hour) per week.	78%
Dimer et al. [51]	Indigenous Australians, Metropolitan area, Western Australia, Australia; Cohort, *n* = 98 (females *n* = 63, males *n* = 35); Mixed gender, age: 55 ± 13 (19–82).	Build it and they will come: outcomes from a successful cardiac rehabilitation program at an Aboriginal medical service. Evaluate the uptake and effects on lifestyle, and cardiovascular risk factors, of cardiac rehabilitation at an Aboriginal Medical Service.	Exercise prescription and education sessions over 8 weeks Stationary cycling and dumbbell exercises were prescribed and supervised. An outdoor walking group was implemented and participants were asked to record their activity levels. Education sessions included diet, nutrition, risk factor modification and medication usage.	29%
Egger et al. [42]	Torres Strait Islanders, Australia; Cohort, *n* = 47; Male only, age: 41 years ± 12.3 years.	Abdominal obesity reduction in indigenous men. To decrease bodyweight and body fat in Indigenous males in the Torres Strait Islands.	There were four lifestyle messages for the program, which included: reducing fat intake, increasing dietary fibre, increasing daily movement, and changing obesogenic habits. The program was delivered in groups via audio-taped conversations.	66%
Gracey et al. [52]	Aboriginal Australians, Remote Western Australia, Australia; Cohort, *n* = 416; Mixed gender, age: 38.7 ± 14.9 [18–82years] (male), 41.0 ± 17.4 [18–88years] (female).	An Aboriginal-driven program to prevent, control and manage nutrition-related “lifestyle” diseases including diabetes. To heighten awareness about lifestyle diseases and promote healthier living through better diet and regular physical activity.	Increase awareness of and promotion of healthier living through better nutrition and the encouragement of regular exercise, sports and active recreation.	Not specified
McAuley et al. [36]	Māori, New Zealand; Cohort, *n* = 36 (female *n* = 28, male *n* = 8); Mixed gender, age: 41.3 years [24–60].	Implementation of a successful lifestyle intervention programme for New Zealand Māori to reduce the risk of type 2 diabetes and cardiovascular disease. To reduce the risk of type 2 diabetes and cardiovascular disease.	Participants were prescribed individual diet and exercise programs. In addition, participants were invited to exercise sessions four times per week and a healthy food sessions once a month in the form of a cooking group.	86%
Mendham et al. [41]	Indigenous Australians, Regional New South Wales, Australia; RCT, *N* = 26 (control *n* = 10, intervention *n* = 16); All male, age: 39.5 ± 10.6 years (intervention), 36.1 ± 16.1 years (control).	A 12-week sports-based exercise programme for inactive Indigenous Australian men improved clinical risk factors associated with type 2 diabetes mellitus. To assess changes in clinical risk-factors following a 12-week exercise program.	Supervised group-based cardiovascular and resistance exercises were conducted at a local fitness centre over 12 weeks.	Intervention = 41% Control = 63%
O’Dea [39]	Indigenous Australians, Remote Western Australia, Australia; Cohort, *n* = 14 (diabetics *n* = 10, non-diabetics n = 4); Mixed, age: 59.3 ± 1.8 years (diabetics), 52.3 ± 4.3 years (non-diabetic).	Marked improvement in carbohydrate and lipid metabolism in diabetic Australian Aborigines after temporary reversion to traditional lifestyle. Improve all aspects of carbohydrate and lipid metabolism that are linked to insulin resistance after temporary revision to traditional lifestyle.	Participants were taken to a remote location and lived a hunter/gatherer lifestyle for 7 weeks.	100%
Rowley et al. [35]	Aboriginal Australians, Remote Western Australia, Australia; Cohort, *n* = 96; Mixed gender, age: 49 ± 3 years (intervention), 43 ± 4 years (control).	Effectiveness of a community-directed ‘healthy lifestyle’ program in a remote Australian aboriginal community. Assess the sustainability and effectiveness of a community-directed program for primary and secondary prevention of obesity, diabetes and cardiovascular disease in an Aboriginal Community.	Formal and informal education sessions about nutrition, regular physical activity sessions such as hunting groups, sports (2–3 sessions) and walking groups (3–4 times per week, for an hour) and walking groups.	51%
Sukala et al. [53]	Polynesian (New Zealand Māori, Cook Island Māori, Samoan, Fijian, Tokelauan & Tongan), Porirua, New Zealand; Cohort, *n* = 18 (females *n* = 13, males *n* = 5); Mixed genders, age: 49 ± 5 years.	Exercise improves Quality of Life in Indigenous Polynesian peoples With type 2 diabetes and visceral obesity. The aim of the study was to evaluate the differential effects of 2, group-based exercise modalities on quality of life (QoL) in indigenous Polynesian peoples with type 2 diabetes (T2DM) and visceral obesity.	Intervention included 3 exercise sessions per week (40–60minutes), consisting of resistance training and aerobic training.	69%

**Table 3:** Outcome measures, impact and intervention development and duration

	Objective outcome measures	Subjective outcome measures	Metabolic measures	Data collection points, duration of intervention	Cultural consultation and adaption
Biddle et al. [48]	Vo2 peak**↑, leg strength (maximal quadriceps at 60deg/second)*↑	PAR-Q	Fasting glucose and glycated haemoglobin (HbA_1c_), lipid profile (HDL)*↑, blood pressure and C-reactive protein	Baseline, 4 weeks, IP	Community consultations
Canuto et al. [49]	Height, weight*↓, BMI*↓, waist and hip circumference, and blood pressure, Step count [data not shown]	Sallis seven-day physical activity recall survey (1985), Quality of life (SF36)	Fasting venous samples of glucose and serum insulin, total cholesterol, high-density lipoprotein (HDL) and triglyceride concentration. HbA_1c_ and c-reactive protein	Baseline, 12 weeks, IP, 12WP,	Community consultations
Chan et al. [43]	Weight, Waist*↓ and hip circumference, Blood pressure*↓, Step count		Plasma glucose (fasting), HbA_1c_*↓, Lipid profile*↓ (LDL cholesterol, HDL cholesterol, triglycerides*↓, Homocysteine, C-peptide, Serum creatinine, Microalbuminuria, Insulin resistance, Triglyceride, Homocysteine levels	Baseline, 6 months	Community consultations
Coppell et al. [50]	Weight, waist circumference, blood pressure*↓, BMI	Medical history, self-reported physical activity*↑ and dietary behaviours**↑↓ (New Zealand Health Survey, 1999).	75g fasted oral glucose tolerance test (OGTT)*↑, Fasting insulin, fasting lipids ↓*and urate*↓ and a mid-stream urine sample*↑.	Baseline, ongoing, two years post intervention	Community consultations
Davey et al. [40]	Age, gender, health conditions, Weight*↓, BMI*↓, waist circumference *↓, 6 minute walk test (6MWT)*↑, Incremental shuttle walk test (ISWT)*↑, Timed Up and Go (TUG)*↓	Chronic Respiratory Questionnaire (CRQ), Quality of life (SF36)*↑		Baseline, 8 weeks, IP	Community consultations
Dimer et al. [51]	Weight, BMI*↓, 6 minute walk test (6MWT)**↑, Blood pressure*↓, Waist girth**↓			Baseline, 8 weeks, IP	Community consultation
Egger et al. [42]	Weight**↓, Waist & hip circumference (cm)**↓, BMI**↓, hip circumference **↓, Waist to hip ratio, Body fat through bio-impedance analysis**↓., fat mass (kg)**↓			1 year follow up	Community consultation
Gracey et al. [52] †	Weight, BMI, Blood pressure		HbA_1c_, glucose, cholesterol (total, LDL, HDL), triglycerides	Not specified	Community consultation
McAuley et al. [36]	Weight**↓, height, waist and hip circumference**↓, blood pressure*↓, BMI**↓, Body composition (fat free mass)*↓, and submaximal exercise test (one mile walk test)**↑	4 day diet record pre and post intervention and a daily diet record. *↓ PAR-Q Physical activity levels: Based on the Life in New Zealand (LINZ)	fasting insulin, glucose**↓, and lipids, insulin sensitivity**↓	Baseline, 4 months, IP	Community consultation
Mendham et al. [41]	Body mass*↓, blood pressure, waist circumference*↓ (WC) and hip circumference, BMI*↓ A graded exercise test (GXT)*↑ to determine peak oxygen consumption (VO2 peak) and maximal aerobic workload (*W*max)	PAR-Q	Leptin (pg mL^−1^)**↓, Glucose regulation: Fasting glucose test and oral glucose tolerance test (OGTT), Inflammatory markers: C-reactive proteins and inflammatory cytokines. Matsuda ISI (μlUmL^−1^, mg mL^−1^)*↑, HOMA-IR (μlUmL^−1^, mg mL^−1^)*↑	Baseline, 12 weeks, IP	Community consultation
O’Dea [39]	Weight, BMI	Physical activity levels on a scale of 1–5.	Oral glucose tolerance test*↓, fasting plasma insulin *↓Plasma triglycerides*↓ , plasma cholesterol Fasting plasma glucose*↓	Baseline, 7 weeks, IP	Unsure
Rowley et al. [35]♦	Body weight, BMI**↓	Diet and physical activity questionnaires [not specified]	75g oral glucose tolerance test (OGTT)**↓, Fasting plasma triglyceride*↓ and insulin concentrations*↑ Fasting plasma glucose *↓	Baseline, 6M, 12M, 18M, 24M, 48M	Community consultation
Sukala et al. [53]	Weight, BMI, Blood pressure	SF36 (Quality of Life)*↑		Baseline, 16 weeks, IP	Community consultation

^*IP* immediate post intervention, * Significant change (*p*<0.05), ** Significant change (*p*<0.01), ↓Value decreased, ↑Value increased, † Not enough information provided to determine significant differences, ♦Significance level was set at *p*<0.1^

## Results

Through database searching 407 records were retrieved, all of which were published in English. Five additional records were identified through backward [39] and forward citation tracking [40–43] and three through searching the grey literature. After removing duplicates, 287 records were screened and 17 articles were initially considered eligible. Reasons for the exclusion of articles included studies that were based on animal research, were not conducted in Australia or New Zealand or did not have any physical activity components, e.g. a nutrition only intervention. After further consideration, the three studies from the grey literature were excluded due to insufficient information on, and lack of, comparability of pre and post measures of physical activity or health outcomes [44–46]. The grey literature included the Green Prescriptions Patient Survey 2014, which is a report on a study in New Zealand that monitors key performance indicators of health, including changes to physical activity [45]. An Australian study on a lifestyle program called Ngawa Kurumutamuwi (We Are Strong) was excluded due to lack of details on population demographics and statistical analysis [46]. A pilot study on a team-based weight loss competition in Aboriginal communities from the New South Wales Ministry of Health was excluded due to lack of details on the baseline and post measurements [44]. One study from the peer-reviewed literature was excluded because of the overlap of data with another included study [47], leaving 13 studies [35, 36, 39–43, 48–53] in the synthesis. Three studies were published before 2000 [35, 39, 42], four between 2000–2010 [36, 43, 50, 52] and six between 2011 and 2015 [40, 41, 48, 49, 51, 53]. There were three randomised controlled trials eligible for inclusion in the review [41, 48, 49], whilst the remaining studies used cohort designs [35, 36, 39, 40, 42, 43, 51–53] and an interrupted time series [50].

Figure 1 shows the flow diagram of the study selection process.

### Appraisal of Study Quality

Using the Quality Assessment Tool for Quantitative Studies [38], the overall study quality was rated as poor in three studies [35, 48, 50], moderate in nine [36, 39–43, 49, 51, 52] and strong in only one study [53]. Overall scores for each quality rating item are presented in Table 3. Study selection bias had the weakest score overall, as nine studies were classified as weak due to not being representative of the target population and only one study [52] was classified as strong. No study rated weak in study design or data collection methods, while confounding bias and blinding were not applicable to the majority of the studies. In data collection methods, all studies rated strong due to utilising validated and reliable measures for health outcomes and/or physical activity levels.

### Population

All studies were focused on Indigenous adults aged 18 years and over. Nine of the 13 studies were based in Australia and four in New Zealand. The number of participants varied from 14 [39] to 418 in a cohort study that involved four different Aboriginal communities [52]. Seven of the studies had 50 or more participants [35, 40, 43, 49–52]. Locations of the interventions varied from metropolitan, urban and regional areas, [36, 40, 41, 43, 48, 49, 51, 53] to rural and remote locations [35, 39, 42, 50, 52]. Several studies were designed for a specific ethnic group such as Aboriginal Australians [35, 40, 52], Torres Strait Islanders [42], and New Zealand Māoris [36, 50]. In other interventions different ethnic groups were combined such as Indigenous Australians [39, 41, 43, 49, 51], Pacific Islanders [48] and Polynesians [53]. Ten studies had male and female participants, one study exclusively targeted women [49] and for two studies only male participants were recruited [41, 42]. The mean age of participants across all studies ranged from 34 to 55 years and with 18–88 [52] and 19–82 years [51], two studies had very wide participant age ranges.

### Recruitment and Retention of Participants

The intervention programs varied by the recruitment strategies, and the nature and delivery methods of the physical activity component. Some studies had an open invite for all community members [52], recruited through a health centre or patient register [40, 50, 53] or utilised referrals through community leaders or other participants [36, 40, 41, 43, 48, 51]. In some studies it was unclear how participants were recruited [35, 39, 42], and for two studies [36, 49] protocol papers were published which described the methodology in greater detail [54, 55].

Almost half of the studies either did not report information on withdrawals from the study and drop-outs from the physical activity intervention [52] or had less than 60% of participants complete the study or failing to have complete data sets [35, 41, 49–51]. Three studies had a retention rate of participants to the program of 60–79% [40, 43, 53] and for four it was 80% or more [36, 39, 42, 48]. Six of the studies had high numbers of participants lost to follow up, and in one of these studies the drop-out rates from the program or failure to attend follow up data collection were so high that it resulted in the under powering of some of the statistical analysis [49]. Two studies were statistically underpowered in some outcome measures despite moderate to high retention rates [43, 48].

### Study Designs and Interventions

Three studies were randomised controlled trials (RCTs); two were of 12 week exercise interventions [41, 49] and one was a shorter 4 week intervention which consisted of small sided (small number of players per team) team sports [48]. Another study attempted to run an RCT to examine the effect of community directed lifestyle programs on the primary and secondary prevention of obesity and chronic disease, but used a self-selected control group and therefore was not a true randomised controlled trial [35]. An additional study had participants share information on the nature of the diet and exercise lifestyle intervention and became a cohort study, as the control group was integrated into the intervention [36]. One study utilised an interrupted time series design to examine the effect of health promotion and education on high risk individuals to reduce insulin resistance [50] and the remaining seven [39, 40, 42, 43, 51–53] were cohort studies. Some cohort studies measured data pre and post intervention [39, 40, 51, 53], while two studies measured data periodically [42, 43] and one study did not specify when data collection occurred [52].

Ten studies focussed on prevention or management of chronic diseases, such as type 2 diabetes and heart disease [35, 36, 40–43, 50–53], one study focused on cardiorespiratory fitness and health [48] and Canuto et al. [49] evaluated the effectiveness of a 12 week exercise and nutrition program on waist circumference, weight and biomedical markers in Australian Indigenous women. The main objective in the study by O’Dea [39] was to improve clinical risk factors linked to insulin resistance.

Most of the studies included facilitated exercise sessions [35, 36, 40, 41, 48–51, 53]. The modality of exercise sessions varied between the studies. For example, while most studies incorporated mixed aerobic and resistance training programs two to six times per week [36, 40, 41, 49–51, 53], other studies used hunting groups [35] or small-sided (small number of players per team) team sports [48]. In one study physical activity was encouraged through self-monitoring with a pedometer and participants were asked to record their daily step counts [43]. Other studies encouraged physical activity through education and lifestyle messages [42, 52] and in one study participants were taken to a remote location to live a hunter-gatherer lifestyle for seven weeks [39].

Cultural adaptations were noted in all studies. Specific efforts to make programs culturally appropriate included consultation with community members such as elders or an advisory group [36, 49]. Examples of cultural adaptations for interventions were using appropriate local dialect in promotion material [36, 42, 49, 50], using traditional games or historically important cultural activities such as paddling [36] and hunting [35, 39].

### Effects on Physical Activity Levels

Although all studies evaluated an intervention with physical activity as a single or core component, only six studies assessed physical activity via subjective or objective measures before and after the intervention [35, 36, 39, 43, 49, 50], with significant increases in only one study [50]. Two studies employed an objective measure of physical activity (pedometers) [43, 49] and four studies utilised self-reported measures. Self-reported physical activity was measured with a variety of tools such as the Life in New Zealand questionnaire [36] and a validated seven-day recall questionnaire [49]. The variation in the measurement of physical activity levels across studies resulted in data being represented in two older studies as categorical (exercise intensity) [35, 39] and continuous (step counts, or minutes of self-reported physical activity per week) [43, 49], thus making them incomparable. Five studies [36, 40, 41, 48, 51] used tests of functional or exercise capacity and aerobic fitness as outcome measures, and all found significant improvements.

### Effects on Health Outcomes

Nine studies collected metabolic markers, including fasting glucose, insulin, cholesterol and oral glucose tolerance tests [35, 36, 39, 41, 43, 48–50, 52]. Significant improvements were found in seven of these studies [35, 36, 39, 41, 43, 48, 50]. Five of the seven studies that assessed lipid profiles reported significant improvements [35, 39, 43, 48, 50]. In regards to diabetes related markers, HbA_1c_ was measured in three studies [43, 48, 49], but a decrease was only found in one of them [43]. All three studies that used an oral glucose tolerance test reported significant decreases in glucose levels [35, 39, 50]. Three studies measured quality of life with the SF-36 survey [40, 49, 53], with improvements reported in two of the three studies [40, 53] and both studies that reported dietary behaviours had significant positive changes with increased intake of dietary fibre and wholegrain [36, 50] and reduced saturated fat consumption [36]. There were significant reductions in seven of the 12 studies that assessed weight and/or BMI [35, 36, 40–42, 49, 51]. No significant reductions or increases in weight/BMI were reported in four studies [39, 43, 48, 50, 53] and one study did not provide enough information to determine if there were statistically significant differences [52]. Of the eight studies that assessed blood pressure, half had significant reductions in systolic and/or diastolic pressure [36, 43, 50, 51] while the other four did not report significant changes [41, 49, 52, 53].

### Long Term Effects on Physical Activity Levels and Health Outcomes

Seven of the 13 studies only collected follow-up data immediately post intervention [36, 39–41, 48, 51, 53]. In one study the follow-up period was not specified, but only referred to as “several months” after the intervention [52] and two other studies collected follow up data three [49], and six months [43] after the intervention. Only three studies collected long-term follow-up data (12 months or more) [35, 42, 50]. Long term follow-up data collection periods were one [42], two [50] and four years [35]. There were mixed results in regards to maintenance of changes in health outcomes. Canuto et al. [49] implemented a 12 week program and reported modest reductions in weight, BMI and blood pressure immediately post program and 12 weeks later. There were no significant changes in waist circumference and metabolic markers at follow up, but the authors noted that this could be due to a low participant retention rate, which underpowered some of the study results. The intervention group had reductions in median weight from baseline (81.8kgs) to immediately post intervention (80.2kgs), but had gained weight back at the 12 week follow up (81.7kgs). The waitlisted group had an increase in weight over all data collection points prior to receiving the intervention (90.6kgs, 93.2kgs and 95.2kgs respectively). Rowley and colleagues [35] followed participants up during a four year uncontrolled community intervention with mixed results. The program achieved significant reductions in fasting insulin concentrations at 6, 12 and 18 months. At further data collection points (two and four years) fasting insulin concentrations continued to drop significantly in the older age group (35 years and over), but in the younger participants (15 to 34 years), there was no statistically significant reduction over time. There were no changes in the prevalence of overweight or obesity among the older participants, and among younger participants weight increased over time as seen in the BMI data collected at baseline (22.6 kg/m^2^), two years (24.3 kg/m^2^) and four years (24.8 kg/m^2^) (*p* for trend = 0.028). Chan et al. reported that six months into a two year intervention there were significant reductions in waist circumference, blood pressure and clinical markers such as HbA_1c_ and cholesterol [43]. However, for this study data were only reported for the first six months of the intervention. A study in New Zealand [50] reported increased physical activity levels and reductions in the prevalence of insulin resistance after two years of the intervention. The most significant changes were in women aged 25–49 years and those who had the highest levels of participation and marked lifestyle changes. In the one year intervention by Egger et al. the Indigenous Australian men who participated were tested at baseline, 6 and 12 months, and showed continuous reductions in bodyweight, waist circumference and a decrease in fat mass at all time points [42].

## Discussion

Despite the high rates of physical inactivity and preventable chronic disease among Indigenous people in Australia and New Zealand, there is a dearth of evaluations of physical activity interventions for these population groups. Moreover, similar to other reviews of interventions for Indigenous people [29, 30, 56], not only did we find just a small number of studies, but also that most of them were not methodologically strong. The lack of RCT designs across studies limits the overall internal validity of the findings, however, study methodology and evaluation issues are not unique to the South Pacific. Similar to our findings, a systematic review on physical activity interventions in American Indian and Alaskan native populations in North America reported that the majority of community-based programs was not rigorously evaluated [29].

The effects of the interventions on physical activity levels were hard to determine. Only two of 13 included studies in this systematic review used objective measures of physical activity (pedometers) [43, 49] and another four studies used self-report measures [35, 36, 39, 50] which limits the validity of the data due to recall and social desirability bias [57], especially as these self-report measures were not specifically designed for this population group. In terms of health outcomes, all but one of the included studies used weight or BMI as an outcome measure and seven of these reported significant reductions. However, it is unclear if these significant improvements in weight or BMI were due to an increase in physical activity alone or changes in energy intake which were not reported. Five studies [36, 40, 41, 48, 51] employed some form of fitness test. Improvements in fitness capacity are a valid indicator of increased physical activity, as regular exercise improves anaerobic and aerobic fitness [58]. If objective measures of physical activity were not available, the use of tests of exercise capacity is appropriate. A functional capacity test, such as the six-minute walk test, can be administered to a variety of population types of various fitness levels and health status and requires little equipment [59].

The results from this systematic review need to be interpreted with caution as in all but one study the methodological quality was rated as moderate or poor which limits their internal and external validity. Methodological issues, such as participant recruitment methods, may have caused selection bias. For example, an open community invitation recruitment [52] is subject to selection bias as volunteers are more health conscious [60] and may lead to a sample that is not representative of the target group. In terms of attrition bias, participants lost to follow up may differ from those who completed the study. Participants who could not be reached at follow up had significantly lower weight and/or waist circumference at baseline [35, 40, 42] compared to the participants that remained in the study. Attrition bias also reduces the sample size, which can result in studies being underpowered and therefore caution needs to be taken when interpreting data [42, 48, 49]. Identified factors that can affect attrition rates were highly mobile young populations [50], work commitments, and needing to care for children [61]. However, most of these issues also affect studies in non-Indigenous populations.

The strength of the intervention study designs varied. It is difficult to assess the effect of confounding variables on the study outcomes as most were uncontrolled and non-randomised which is a limitation of the evidence. Despite the benefits of internal validity of randomised controlled trials, as mentioned before, RCTs may not always be feasible in Indigenous populations because it could be considered to be unethical to recruit participants for control groups [35, 36]. Consultations with community members, leaders and health workers were undertaken prior or during almost all of the interventions in order to tailor the design to suit the community resources and culture. Availability of and access to resources for interventions, such as infrastructure, equipment, recreational facilities and health workers, are influenced by geographical location [62], which in five of the primary studies were in rural or remote communities [35, 39, 42, 50, 52].

Cultural consultations, adaptation and flexibility in the delivery of programs are important for interventions in Indigenous populations. Interventions that included facilitated exercise sessions, as opposed to written information, might be more appropriate for some Indigenous populations with lower levels of literacy. Gracey et al. [52] noted that the older people may be semi or non-literate and the local people were not comfortable with “high English” as it was a second or third language behind their native tongue. In regards to flexibility in interventions, McAuley et al. [36] noted that their lifestyle intervention had to evolve continuously over time to ensure it was still acceptable to the local Māori people. Similarly, Dimer et al. [51] allowed flexible timing of allocations of participant attendance to the program by not creating specific times for sessions in order to comply with Aboriginal ideologies that conflict with tightly regimented, appointment-based systems. Concepts of time may differ between Indigenous and non-Indigenous people due to cultural values. For instance, Indigenous Australians have a ‘here and now’ approach, meaning that important and immediate priorities will be seen regardless of prior commitments [63]. Specific in-depth details of cultural adaptations may not always be available as adaptations may be seen as part of the usual development process in Indigenous-based programs and are not necessarily described [29].

### Gaps and Limitations in the Current Literature and Directions for Future Research

This systematic review provides a much needed insight into the gaps in the current literature on the effect of physical activity interventions on activity levels and health outcomes in this particular population group. A primary limitation of the existing literature is the challenge of undertaking rigorous study designs, specifically in relation to recruiting control groups. Additionally, only three studies reported long term follow-up data. Without long-term follow up information the sustainability of the impact of the intervention in the community cannot be assessed. This has been noted previously in regards to other Indigenous groups around the world where physical activity interventions are not translated into ‘real-world’ practice despite having short term significant effects, communities lacked resources to sustain the programs [56]. Another limitation of most studies in this review was a lack of reporting of methodological details. Moreover, lack of information in the description of the intervention methodology, such as details about cultural adaptation, cost and process of developing the program, creates issues around best practice as it would then be difficult to replicate successful programs. Missing information from studies included sample size calculations and statistical details on adjustment for confounding variables. The inclusion of sustainability plans, cost-benefit analysis [40], and information on community feedback on factors such as barriers to attendance would be beneficial.

### Recommendations for Practice and Research

Future evaluations of interventions aimed at increasing physical activity levels in Australian or New Zealand Indigenous populations would be improved by more rigorous study designs. Validated objective measures of physical activity would be desirable. Using pedometers or accelerometers would increase the validity and comparability of data between studies. Causal pathways for the health effects of regular physical activity levels include increased aerobic capacity and strength, outcomes that other health behaviours, such as changes in dietary behaviour, would not influence. Therefore, if objective measurement of physical activity is not available due to lack of funding, graded exercise testing or functional capacity assessments are low cost options for researchers and communities to examine direct effects of changes in activity levels.

Community consultations are important to include in study protocols. Although this process can be time consuming [64], it is necessary to make the intervention culturally acceptable to the participants. For example, whilst the challenges and appropriateness of having randomised controlled trials can be determined by the community, pragmatic study designs should be considered. A notable example of working around this problem was the study conducted by Canuto et al. [49], which was the only study to utilise a wait-listed control group.

Studies with low retention rates should also provide information on factors which led to people not attending as it may help researchers in the creation of risk management plans. Coppell and colleagues [50] limited the age range of participants for their intervention to 25 years and over as the community often had young adults leave to get further education or employment in larger towns. Increasing the minimum age of participants could possibly reduce the attrition rates in some communities, however, evidence on effects of physical activity in early prevention of chronic disease in young Indigenous people would be desirable. In most studies, the age range of the participants was limited with no studies focused specifically on young adults. While the incidence of type 2 diabetes in young people in Australia has not changed since 2002 [65], the prevalence of type 2 diabetes has significantly increased in Indigenous Australian adults 35 years and older [66]. In New Zealand, the number of people with diabetes has doubled in the past ten years, with 40 new confirmed diagnoses every day [67]. Therefore, earlier prevention and detection would be beneficial and young adults may be an ideal target group for physical activity interventions in primary health care prevention to avoid or delay acquiring chronic diseases and the later complications that typically manifest 15–20 years after diagnosis [68].

Canuto et al. [61] wrote that logistical aspects, such as transport to classes and competing commitments like family obligations, were factors that influenced attendance of their physical activity intervention and concluded that future studies could identify potential barriers with pre-program workshops. To minimise attrition in their intervention Davey et al. [40] organised free transport for their participants to get to the exercise sessions. They found that the majority of the participants used the provided transport and concluded that this might have contributed to retaining participants.

For future studies, cost-benefit analyses would provide useful information on economic feasibility, sustainability and scalability which can help inform best practice for studies that aim to address the needs of vulnerable populations. Therefore, interventions need to be tested and implemented with economically feasible methods [56].

## Conclusions

Despite of the high rates of chronic disease and physical inactivity in Indigenous populations in Australia and New Zealand, only a very small number of evaluations of physical activity interventions for these population groups have been published. Only 13 studies were identified in this systematic review. Due to the lack of validated measures of physical activity in most studies it is unclear how successful interventions are at increasing activity levels in Indigenous adults in Australia and New Zealand. However, there is evidence to support that interventions with elements of physical activity are successful in improving health outcomes such as weight and various clinical markers. Comparisons between studies was difficult as there was a lack of homogeneity in study designs and outcome measures, which may be due to communities instigating intervention adaptations to be tailored towards their individual needs. Validated measures of physical activity and in-depth detail around the cultural consultation phases, end of project feedback, including strengths and weaknesses, and cost-benefit analysis would be useful in guiding best practice for physical activity interventions in Indigenous settings.
